# Associations of anticholinergic burden of medication with cognitive decline and longitudinal brain atrophy in the Alzheimer’s disease spectrum

**DOI:** 10.3389/fnagi.2026.1751326

**Published:** 2026-03-03

**Authors:** Stefan Teipel, Alice Grazia, Oliver Peters, Josef Priller, Anja Schneider, Jens Wiltfang, Claudia Bartels, Björn Hendrik Schott, Frank Jessen, Emrah Duezel, Renat Yakupov, Katharina Buerger, Robert Perneczky, Christoph Laske, Annika Spottke, Michael Wagner, Jonas Peltner, Ingo Kilimann, Britta Haenisch

**Affiliations:** 1 German Center of Neurodegenerative Diseases (DZNE), Rostock/Greifswald, Germany; 2 Department of Psychosomatic Medicine, University Medicine Rostock, Rostock, Germany; 3 Institute of Psychiatry and Psychotherapy, Charité – Universitätsmedizin Berlin, Berlin, Germany; 4 Department of Psychiatry and Psychotherapy, Charité, Berlin, Germany; 5 Clinic of Old Age Psychiatry and Cognitive Disorders, University Hospital Bonn and University of Bonn, Bonn, Germany; 6 German Center for Neurodegenerative Diseases (DZNE), Bonn, Germany; 7 German Center for Neurodegenerative Diseases (DZNE), Goettingen, Germany; 8 Department of Psychiatry and Psychotherapy, University Medical Center Goettingen, University of Goettingen, Goettingen, Germany; 9 Department of Psychiatry and Psychotherapy, Otto-von-Guericke University, Magdeburg, Germany; 10 Department of Psychiatry, Faculty of Medical, University of Cologne, Cologne, Germany; 11 German Center for Neurodegenerative Diseases (DZNE), Magdeburg, Germany; 12 Institute of Cognitive Neurology and Dementia Research (IKND), Otto-von-Guericke University, Magdeburg, Germany; 13 Department of Radiology, Ludwig Maximilian University Hospital, Munich, Germany; 14 German Center for Neurodegenerative Diseases (DZNE, Munich), Munich, Germany; 15 Department of Psychiatry and Psychotherapy, University Hospital, LMU Munich, München, Germany; 16 German Center for Neurodegenerative Diseases (DZNE), Tübingen, Germany; 17 Section for Dementia Research, Hertie Institute for Clinical Brain Research and Department of Psychiatry and Psychotherapy, University of Tübingen, Tübingen, Germany; 18 Research Division, Federal Institute for Drugs and Medical Devices, Bonn, Germany; 19 Center for Translational Medicine, Medical Faculty, University of Bonn, Bonn, Germany

**Keywords:** Alzheimer’s disease, cholinergic basal forebrain, hippocampus, MRI, treatement

## Abstract

**Background:**

Anticholinergic side effects of pharmacological treatment are a risk factor for cognitive decline in older people. Here, we aimed to assess the effect of anticholinergic burden of treatment on longitudinal rates of cognitive change and atrophy in functionally related brain regions in people from the Alzheimer’s disease (AD) spectrum.

**Methods:**

We determined associations of anticholinergic burden of pharmacological treatment with rates of global cognition, episodic memory and executive function decline as well as basal forebrain and hippocampus atrophy in participants of the memory clinic based DELCODE cohort, spanning the range from cognitively normal through subjective cognitive decline, mild cognitive impairment and AD dementia. We had 794 cases with neuropsychological outcomes, and a subset of 703 cases with MRI outcomes. Effects were assessed using mixed effect models in a Bayesian framework using prior-insensitive cross-validated Bayes factors (CV-BF) and parameter estimates.

**Results:**

We found moderate evidence for an association of anticholinergic burden with baseline levels of cognitive impairment for the PACC5 as a global cognitive function score (CV-BF = 9.0) with more impairments with higher burden, but not with basal forebrain and hippocampus volumes, and weak evidence for an association of anticholinergic burden with longitudinal rates of change in the trail-making test B as an executive function score (CV-BF = 2.5), but not for other cognitive scores and not for brain volumes.

**Conclusion:**

In the presence of prodromal or manifest AD, in a memory clinic-based cohort anticholinergic burden had only a modest effect on cognitive decline and no effect on atrophy in brain regions that are related to the cholinergic system.

## 1 Introduction

Anticholinergic side effects of pharmacological treatment are a risk factor for cognitive decline and delirium in older people (Dauphinot et al., 2017; Margolis et al., 2021; Pistorio et al., 2025), however, with a limited level of evidence (Taylor-Rowan et al., 2021). Anticholinergic burden is a widely adopted prognostic factor to estimate future clinical outcomes in older adults (Taylor-Rowan et al., 2021). It is typically assessed by assigning scores to medications and calculating a cumulative total, but existing measures vary considerably in the drugs included, the scores assigned, as well as the dosage and duration of consumption (Taylor-Rowan et al., 2021). Previous studies have reported cross-sectional brain atrophy and impaired cognitive performance associated with the anticholinergic burden of treatment in older people with and without neurocognitive disorders (Kilimann et al., 2022; Malkiewicz et al., 2023). Similarly, longitudinal studies have shown that anticholinergic burden can predict global cognitive decline over time in older adults (Brombo et al., 2018; Broder et al., 2022), as well as memory (Papenberg et al., 2017; Moriarty et al., 2021) and executive dysfunctions (Risacher et al., 2016).

Here, we built on previous research by testing the hypothesis that the anticholinergic burden of medication is associated with faster rates of cognitive decline and regional brain atrophy in individuals across the Alzheimer’s disease (AD) spectrum, including those with normal cognition, subjective cognitive decline, mild cognitive impairment, and dementia (Jessen et al., 2018). Our analysis focused on the cholinergic basal forebrain as the main source of neocortical and allocortical acetylcholine (Schliebs and Arendt, 2011), the hippocampus as an important target region for cholinergic projections (Selden et al., 1998), and executive and memory functions that are strongly linked to the integrity of the cholinergic system (Peter et al., 2016; Nemy et al., 2020). This was done on the assumption that these brain regions and the related cognitive functions are particularly vulnerable to the anticholinergic side effects of treatment. We employed a Bayesian framework to estimate evidence both for and against an effect of anticholinergic burden. Our results will have important clinical implications, given that 20–50% of older adults are prescribed such drugs worldwide (Fox et al., 2014). In Germany, the prevalence is between 38 and 54% (Kruger et al., 2021).

## 2 Materials and methods

### 2.1 Participants

We used data of the multicenter DELCODE study, including 1,079 cases at baseline, conducted by the German Center for Neurodegenerative Diseases (DZNE) (Jessen et al., 2018). We excluded cases without available MRI scans, cognitive testing or anticholinergic burden score at baseline and no MRI follow up, leaving 703 cases. When only considering cognitive outcomes, we had a larger sample of 794 cases. The sample group consisted of older healthy controls, first-degree relatives of a person with a documented diagnosis of AD dementia, and participants with AD dementia (ADD), MCI or subjective cognitive decline (Table 1). DELCODE excluded participants with a current major depressive episode, past or present major psychiatric disorders, neurological diseases other than AD, or unstable medical condition (Jessen et al., 2018). Subjective cognitive decline (SCD) was defined as a persistent self-perceived cognitive decline in the absence of objective cognitive impairment as measured by the CERAD test battery, lasting at least for 6 months and being unrelated to an acute event (Jessen et al., 2014). The MCI patients met the core clinical criteria for MCI according to National Institute on Aging-Alzheimer’s Association (NIA-AA) workgroup guidelines (Albert et al., 2011). The ADD patients had a clinical diagnosis of probable ADD according to the NIA-AA workgroups guidelines (McKhann et al., 2011). The control participants and the first-degree relatives had no objective cognitive impairment in cognitive tests, no history of neurological or psychiatric disease and did not report self-perceived cognitive decline. All participants or their representatives provided written informed consent. The study protocol was approved by the local institutional review boards and ethical committees of the participating centers. It was conducted in accord with the Helsinki Declaration of 1975.

**Table 1:** Participants’ demographics (cases with longitudinal MRI).

	CN (*n* = 177)	Relatives (*n* = 63)	SCD (*n* = 294)	MCI (*n* = 105)	ADD (*n* = 64)
Sex (female/male)^1^	97/80	35/28	134/160	46/59	35/29
Age (mean, 95% SD) [years]^2^	69.2 (5.3)	66.6 (4.7)	71.0 (6.1)	73.0 (5.7)	74.8 (6.4)
Education (mean, 95% CI) [years]^3^	14.8 (2.8)	14.8 (2.8)	14.9 (3.0)	14.1 (3.1)	12.5 (3.1)
MMSE score (mean, 95% CI)^4^	29.5 (0.8)	29.6 (0.7)	29.3 (1.0)	28.2 (1.5)	22.5 (3.6)
ACB sum score (range 0 to 4)^5^	0.2 (0.5)	0.14 (0.5)	0.5 (0.9)	0.4 (0.8)	0.5 (0.9)
Follow-up (median, 25 and 75% quantile) [years]^6^	4.1 (3.1–5.1)	3.1 (1.3–4.1)	3.0 (2.1–4.1)	3.0 (1.3–4.1)	2.1 (1.1. – 3.1)

CI = credible interval, acb = anti-cholinergic burden, CN = cognitively normals, Relatives = first degree relatives of people with dementia, SCD = Subjective Cognitive Decline, MCI = Mild Cognitive Impairment, ADD = Alzheimer’s disease dementia, MMSE = Mini-Mental State Examination.

^1^Bayes factor was strongly in favor of no dependence between diagnosis and sex (BF_10_) = 0.010; i.e., the independency between sex and diagnosis is 1/0.01 = 100 times more likely than the dependence of both factors.

^2^Bayes factor extremely in favor of a group effect (BF_10_) = 4.7 * 10^14^.

^3^Bayes factor extremely in favor of a group effect (BF_10_) = 5.5*10^4^.

^4^Bayes factor extremely in favor of a group effect (BF_10_) = 6.5*10^150^.

^5^Bayes factor indicates an weak level of evidence in favor of a group effect (BF_10_) = 2.6.

^6^Bayes factor extremely in favor of a group effect (BF_10_) = 6.3*10^14^.

### 2.2 Neuropsychological assessment

We used the delayed recall of logical memory of the Wechsler Memory Scale-Revised as a measure of memory function, the Wechsler Memory Scale-Revised digit span (average of digit span forward and backward) as measure of working memory, and the Trail Making Test B as measure of executive function (Arbuthnott and Frank, 2000). For assessing global cognitive decline, we used the Preclinical Alzheimer’s Composite with Semantic Processing (PACC5) score and the Clinical Dementia Rating (CDR) total score. The PACC is a multi-domain cognitive composite that includes measures of processing speed, global cognition, and memory. It was originally constructed to emphasize memory, as this is a core domain that declines in AD. The PACC5 is an optimized version of the PACC that includes the same measures plus a Semantic Fluency measure. This new version was developed after emerging evidence suggested that semantic fluency declines earlier in the AD trajectory than previously hypothesized and provides unique information about Aβ-related memory changes (Papp et al., 2017). The CDR is a global dementia rating scale that assesses cognitive change, determines the presence of dementia, and quantifies dementia severity from very mild (CDR 0.5) to mild (CDR 1), moderate (CDR 2), and severe (CDR 3). CDR evaluates cognitive and functional performance across six domains (memory, orientation, judgment and problem solving, community affairs, home and hobbies, and personal care), providing an overall index of dementia severity (Williams et al., 2013).

### 2.3 Determination of anticholinergic burden score

The German anticholinergic burden score (Kiesel et al., 2018) was used to identify medications with anticholinergic effects used by the participants. Many of the published scales that measure anticholinergic burden originate from England, the US or Australia. However, drugs authorised in these countries can differ from those authorised in Germany. This is why it was important to use a score tailored to drugs commonly used and authorised in Germany, to ensure all relevant medications were included in the calculation of anticholinergic burden and it was not underestimated. Like other anticholinergic burden scores the German anticholinergic burden score categorizes drugs as having no anticholinergic effects (score = 0), weak anticholinergic effects (score = 1), moderate anticholinergic effects (score = 2), and strong anticholinergic effects (score = 3). Scores for drugs available on the German market were determined by a systematic literature review of existing anticholinergic burden scores and subsequent expert discussion. The total anticholinergic burden score of a patient is the sum of the anticholinergic burden scores of all medications taken by a patient. The burden score was based on self-reporting and obtained annually at each clinical visit; for the current analyses, we used burden scores assessed at baseline.

### 2.4 MRI acquisition

MRI data were acquired from nine Siemens 3.0 Tesla MRI scanners (4 Verio, 1 Skyra, 3 TimTrio and 1 Prisma system) using identical acquisition parameters and harmonized instructions. To ensure high image quality throughout the acquisition phase, all scans had to pass a semi-automated quality check during the study conduction, so that protocol deviations could be reported to the study sites, and the acquisition at the respective site could be adjusted. High-resolution T1-weighted anatomical images were obtained using a sagittal magnetization-prepared rapid gradient echo (MPRAGE) sequence (field of view 256 × 256 mm, matrix size 256 × 256, isotropic voxel size 1 mm, echo time 4.37 ms, flip angle 7°, repetition time 2,500 ms, number of slices 192, parallel imaging acceleration factor 2). The duration of the sequence was 5 min 8 s.

### 2.5 Imaging data processing

The T1-weighted anatomical images were initially coregistered to the mean functional images and subsequently preprocessed using the Computational Anatomy Toolbox (CAT12, v9.6/r7487) (Kurth et al., 2015) for Statistical Parametric Mapping 12 (SPM12, v12.6/r1450, Wellcome Centre for Human Neuroimaging, London, UK). The images were segmented into grey matter (GM), white matter (WM) and CSF, followed by spatial normalization to the default CAT12 brain template in Montreal Neurological Institute (MNI) reference space using the Diffeomorphic Anatomical Registration Through Exponentiated Lie Algebra (DARTEL) algorithm. During this step, the images were resliced to an isotropic voxel size of 1.5 mm, and modulated to adjust for expansion and shrinkage of the grey matter tissue. We then applied a mask containing the cholinergic nuclei of the basal forebrain (Kilimann et al., 2014) to derive the raw basal forebrain volumes. For hippocampus volumetry, we used the harmonized hippocampus segmentation protocol, an internationally driven effort under the auspices of the Alzheimer’s association (Frisoni et al., 2015), implemented into an automated volumetry pipeline to ease processing of larger numbers of cases (Wolf et al., 2017). The raw volumes were divided by the total intracranial volume to adjust for head size. The resulting normalized basal forebrain and hippocampus volumes were entered in the statistical models.

### 2.6 Statistical analysis

Demographic characteristics were compared between diagnostic groups using Bayesian ANOVA and contingency tables as appropriate. We used *Jeffreys’ Amazing Statistics Program* (JASP Version 0.19.3.0), available at jasp-stats.org, to calculate the models. We report the Bayes Factor (BF_10_) quantifying evidence against the null hypothesis (Wagenmakers et al., 2018).

Association of cognitive and volume changes with anticholinergic burden at baseline was determined using Bayesian generalized mixed effects models with time nested within individuals with random slope and intercept terms, and longitudinal cognitive scores or brain volumes as outcomes. Models included the main and the interaction effect of anticholinergic burden with time together with the main and the interaction effect of baseline diagnosis with time, and the main effects of sex, age, and education. We determined fit of individual models using posterior predictive checks. All models were calculated using package “brms” in R, accessed through R Studio (version 2024.12.1).

Inference was conducted in two steps: First, we examined the evidence for or against the alternative model (including the main effect and the interaction effect of anticholinergic burden) versus the null model (excluding these effects), using cross-validated Bayes factors (Hart and Malloure, 2019), see below. Second, we determined the parameter estimates and their 95% credible intervals for the main and interaction effects of anticholinergic burden.

The Bayes factor BF_10_ is the ratio of posterior to prior odds in favor of the alternative model over the null model. Three conclusions are possible within the Bayesian framework (Wagenmakers et al., 2018): support for either the null hypothesis (BF_10_ ≤ 0.33), support for the alternative hypothesis (BF_10_ > 3), or weak evidence (BF_10_ between 0.33 and 3). We applied the following evidence categories: a BF_10_ above 3 provides “moderate evidence,” a BF_10_ above 10 provides “strong evidence,” a BF_10_ above 30 provides “very strong evidence” and a BF_10_ above 100 provides “extreme evidence” against the null model (Wagenmakers et al., 2018).

The use of the cross-validated Bayes factor was motivated by the following rationale: The Bayes factor is independent of the prior probabilities of the two models under consideration (if both are set equal), but it depends on the prior distributions for the parameters of the two models. This leads to high sensitivity of the Bayes factor to the choice of the priors (O'Hagan, 1995), particularly for more complex models such as the mixed effect models assessed here. To overcome this limitation, we first tried sensitivity analyses, where we chose different priors and compared the resulting Bayes factors using bridge sampling with the “bayes_factor” function in the “brms” package in R. However, the results varied greatly depending on the prior distributions chosen for the parameters, so we considered it unreliable to use any of these outcomes. One alternative for assessing model fit is the use of cross-validation. The R library “loo” (Vehtari et al., 2017) provides an efficient leave-one-out cross-validation. It provides an approximation of repeatedly reassessing the model after excluding one observation at a time. However, in longitudinal data, several observations are typically nested within individuals. So leaving just one observation out interferes with the hierarchical structure of the data (Vehtari et al., 2017).

As a potentially powerful alternative, Hart and Malloure proposed a cross-validation Bayes factor (CVBF) that uses an iterative data split and calculates Bayes factors based on the posterior likelihoods from the training sample applied to the validation sample (Hart and Malloure, 2019). This approach is relatively independent of the parameter priors, as their choice influences the calculation of the posterior likelihood in the training sample, however, this is relatively insensitive to the prior distribution of the parameters. Annotated R code for calculating the CVBF using data splits by individuals (respecting the hierarchical structure of the data) is provided as Supplementary material A. We determined CVBF combined with a sensitivity analysis using both a mildly informative prior according to a Gaussian distribution with mean 0 and a standard deviation of 1 (*N* (0, 1)), as well as a moderately informed prior for the model parameters (*N* (0, 0.5)). Numerically, the CVBF can be interpreted in the same evidence categories as the classical Bayes factor.

Second, we determined parameter estimates and their 95% credible intervals to determine strength of effects. We chose a moderately informative prior (*N* (0, 0.5)); this analysis was insensitive to the choice of the parameter priors.

## 3 Results

We identified 703 cases with at least one MRI follow-up scan. The detailed demographic data are reported in Table 1. For sex distribution we found very strong evidence for no difference between groups. Whereas, for age, education, Mini-Mental State Examination (MMSE) score, and length of follow-up we found extreme evidence for a group difference, for anticholinergic burden the evidence was inconclusive (Table 1). As expected, ADD patients were oldest, least educated and had lowest MMSE scores. When we selected participants for availability of at least one follow-up cognitive test, we retrieved 794 cases, including 198 controls, 68 first degree relatives, 345 SCD cases, 130 MCI cases, and 53 ADD patients.

**Figure 1:**
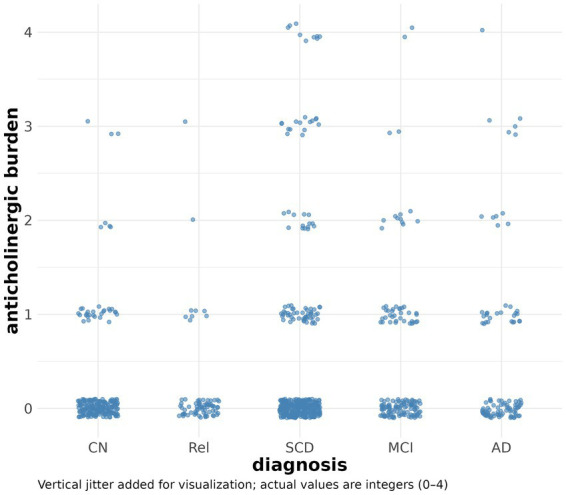
Distribution of anticholinergic burden scores at baseline across diagnoses. Scatter plot of anticholinergic burden scores by diagnosis. For the sake of visualization, the integer anticholinergic sum scores (between 0 and 4) are shown with a jitter.

The anticholinergic burden score was higher in ADD, MCI and SCD cases, compared with controls and first-degree relatives (Figure 1) with moderate evidence for a group difference (BF_10_ = 8.73). However, evidence of an overall effect across groups was inconclusive when controlling for age and sex (BF_10_ = 1.5). *Post hoc* comparisons revealed moderate to strong evidence of a higher anticholinergic burden in ADD and MCI cases compared with controls and first-degree relatives. There was inconclusive evidence of a difference between SCD cases and the other diagnostic groups (Table 2).

**Table 2:** *Post hoc* comparisons for anticholinergic burden by diagnosis.

		Prior odds	Posterior odds	BF_10, U_	Error %
ADD	CN	0.320	23.249	72.764	4.236 × 10^−4^
	MCI	0.320	0.057	0.178	0.060
	Rel	0.320	4.025	12.597	2.436 × 10^−7^
	SCD	0.320	0.112	0.351	0.041
CN	MCI	0.320	15.644	48.962	5.409 × 10^−4^
	Rel	0.320	0.067	0.210	0.055
	SCD	0.320	0.550	1.720	0.012
MCI	Rel	0.320	3.126	9.784	0.003
	SCD	0.320	0.063	0.197	0.083
Rel	SCD	0.320	0.337	1.055	0.016

The posterior odds have been corrected for multiple testing by fixing to 0.5 the prior probability that the null hypothesis holds across all comparisons (Westfall et al., 1997). Individual comparisons are based on the default t-test with a Cauchy (0, r = 1/sqrt (2)) prior. The “U” in the Bayes factor denotes that it is uncorrected.

CN = cognitively normal, Rel = first degree relatives of people with dementia, SCD = Subjective Cognitive Decline, MCI = Mild Cognitive Impairment, ADD = Alzheimer’s disease dementia.

We found moderate evidence for an association of anticholinergic burden with baseline levels of cognitive impairment for PACC5 score with more impairments with higher burden, but not with basal forebrain and hippocampus volumes (Table 3). In the longitudinal analyses, we found weak evidence for an association of anticholinergic burden with longitudinal rates of change in TMT-B score, but not for any other cognitive scores and brain volumes (Table 3). In a sensitivity analysis, we determined if adding ApoE e4 genotype, binarized into no vs. at least one ApoE e4 allele, changed these associations Presence of at least one ApoE e4 genotype had a negative effect on PACC5 score at baseline (estimate −0.11 and 95% credible interval [−0.22 – −0.01]), with preservation of the effect of anticholinergic burden (estimate −0.06 [−0.11 – −0.001]). ApoE E4 genotype had a positive effect on rate of change in TMT-B scores over time (faster decline of function over time with at least one ApoE e4 allele) (estimate 2.38 [1.02–3.75]); again, adding ApoE E4 genotype to the model did not change the interaction effect of anticholinergic burden with time (estimate 1.00 [0.02–1.96]).

**Table 3:** Effects of anticholinergic burden.

Outcome	CV-BF	95% CI lower	95% CI upper
Main effects			
Informed priors (normal (0, 0.5))			
PACC5	9.0	6.3	12.9
CDR-SB	0.23	0.17	0.31
Digit span fw/bw	0.83	0.73	0.95
TMT-B	1.31	1.23	1.39
basal forebrain/TIV	0.47	0.53	0.70
hippocampus/TIV	2.36	1.80	3.05
Mildly informed priors (normal (0, 1))			
PACC5	9.2	6.6	13.1
CDR-SB	0.39	0.30	0.51
Digit span fw/bw	0.72	0.63	0.79
TMT-B	2.60	2.34	2.84
basal forebrain/TIV	0.54	0.47	0.62
hippocampus/TIV	2.02	1.55	2.59
Mildly informed priors (normal (0, 0.5))			
Interaction effects with time			
PACC5	0.96	0.89	1.02
CDR-SB	0.61	0.55	0.67
Digit span fw/bw	0.98	0.91	1.04
TMT-B	2.53	2.23	2.84
basal forebrain/TIV	1.05	0.98	1.12
hippocampus/TIV	0.93	0.8	1.07
Flat priors			
PACC5	0.90	0.84	0.97
CDR-SB	0.82	0.73	0.91
Digit span fw/bw	0.91	0.85	0.97
TMT-B	2.19	1.84	2.64
basal forebrain/TIV	0.88	0.81	0.95
hippocampus/TIV	0.83	0.72	0.96

**Figure 2:**
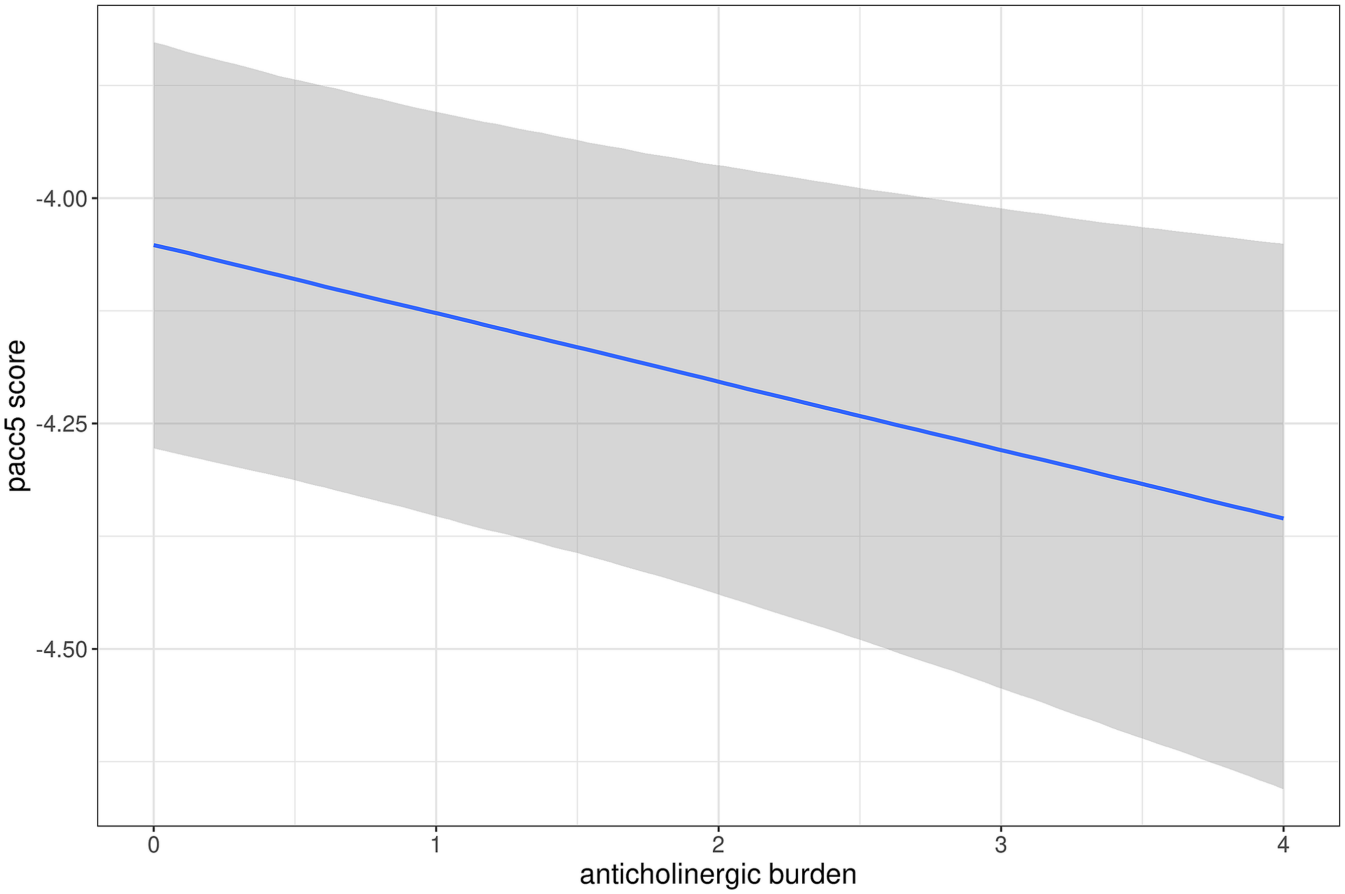
Association of anticholinergic burden with baseline performance in PACC5 scores. Marginal association of anticholinergic burden score with PACC5 scores, controlling for diagnosis, age, and sex. The grey ribbon features the 95% credible interval for the parameter estimate.

**Figure 3:**
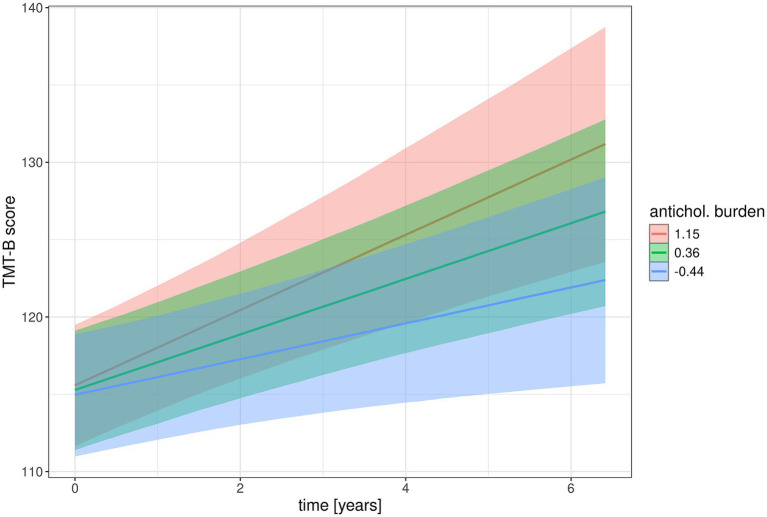
Association of anticholinergic burden with longitudinal change in TMT-B scores. Marginal interaction effects of time with anticholinergic burden score on TMT-B scores as dependent variable in mixed effect models predicting cognitive scores by anticholinergic burden score and diagnosis and their interaction with time as well as age and sex with random slope and intercept terms, nested within individuals. Trajectories feature the 95% credible intervals for estimates of change. TMT-B scores are continuous, here splits are shown for mean TMT-B scores and mean +/− 1 standard deviation.

Parameter estimates for main and interaction effects of anticholinergic burden scores are shown in Table 4, the parameters for all predictors are shown in Supplementary Table 1. The 95% credible intervals excluded zero only for the main effect of anticholinergic burden on PACC5 score and the interaction effect of anticholinergic burden with time on TMT-B, with lower performance with higher anticholinergic burden. Figures 2, 3 show marginal effects of anticholinergic burden on PACC5 scores at baseline and the interaction effect of anticholinergic burden by time on change in TMT-B scores, respectively.

**Table 4:** Parameter estimates for anticholinergic burden main and interaction effects (with mildly informed prior *N* (0, 0.5)).

Outcome	Parameter	Estimate [95% CI]
PACC5	antichol. burden	**−0.0727 [−0.1.306 – −0.0147]**
PACC5	time:antichol. burden	−0.0012 [−0.0188 – 0.0164]
CDR	antichol. burden	0.0873 [0.0082 – 0.1.652]
CDR	time:antichol. burden	−0.0106 [−0.0567 – 0.0354]
digit span total	antichol. burden	−0.0783 [−0.3.072 – 0.1.467]
digit span total	time:antichol. burden	−0.0214 [−0.0937 – 0.0489]
TMT-B	antichol. burden	0.3928 [−0.5.679 – 1.352]
TMT-B	time:antichol. burden	**0.8018 [0.0514 – 1.5491]**
basal forebrain	antichol. burden	−0.0011 [−0.0043 – 0.0021]
basal forebrain	time:antichol. burden	0.0003 [−0.0007 – 0.0013]
hippocampus	antichol. burden	−0.02 [−0.0619 – 0.0219]
hippocampus	time:antichol. burden	0.0027 [−0.003 – 0.0084]

Bold values means numbers exclude zero.

To see the development of anticholinergic burden scores over time, we show individual trajectories of change in anticholinergic burden sum score over time, split according to baseline levels of anticholinergic burden for better visibility. The plot shows that most cases stayed at the initial levels of anticholinergic burden. A smaller fraction showed an increase of anticholinergic burden over time. Only a very small fraction of cases showed moderate decline of anticholinergic burden over time from initial moderate to high levels (Supplementary Figure 1).

## 4 Discussion

Against our hypothesis, we found only limited evidence for an association of anticholinergic burden of treatment with baseline levels and rates of cognitive decline in a cohort spanning the AD spectrum from cognitively normal to dementia. At baseline, anticholinergic burden was associated with levels of cognitive performance in the PACC5 score as measure of global cognition, independently of ApoE e4 genotype, but not with the CDR sum of boxes as a global functional measure, nor with cognitive scores for episodic memory and executive function. The PACC5 score integrates measures of episodic memory, global cognition, semantic fluency, processing speed and executive function (Papp et al., 2017). These components have been found to be related to anticholinergic treatment before (Lopez-Alvarez et al., 2019). A cross-sectional study of a memory clinic sample with 173 cases reported associations of anticholinergic burden higher than 3 (high risk) with the MMSE score as global cognitive measure (Pistorio et al., 2025). As a composite score, PACC5 may be more sensitive to subtle changes than single scores of digit span, however, this presumed advantage was not found in the longitudinal analysis, where anticholinergic burden at baseline was associated with stronger decline in TMT-B performance with a small effect size, independently of ApoE e4 genotype, but not with PACC5 score or any other cognitive test. Based on previous evidence from cross-sectional data (Attoh-Mensah et al., 2020), we had expected an association of anticholinergic burden with steeper decline in executive function as measured by the TMT-B, however, the effect size was small.

Again, in contrast with our *a priori* hypothesis, we did not find an association of anticholinergic burden with basal forebrain and hippocampus volumes. This is partly consistent with a previous study on the UK biobank data of about 17,000 healthy middle-aged and older individuals that reported associations with baseline cognition but not with brain atrophy (Mur et al., 2023). Our findings are in contrast to an analysis of ADNI data that found reduced brain volumes at baseline as well as higher risk of progression to MCI or ADD in people with anticholinergic medication (Risacher et al., 2016). Another analysis of ADNI data found reduced basal forebrain volume in MCI cases with anticholinergic burden, but not in cognitively normal people (Meng et al., 2022). In contrast to the two previous ADNI studies (Risacher et al., 2016; Meng et al., 2022), the majority of our participants were people with subjective cognitive decline, representing a very early stage of cognitive aging and prodromal AD (Jessen et al., 2014). SCD cases may have more preserved cholinergic basal forebrain volume and function and therefore be less vulnerable to anticholinergic side effects of treatment than MCI or dementia stages of AD. Lastly, the current findings are in contrast with a previous population-based study we conducted which comprised healthy adults from Northeastern Germany living in Western Pomerania (Kilimann et al., 2022; Malkiewicz et al., 2023). There we found a statistically significant inverse association between the ACB sum score and the hippocampus volume (Kilimann et al., 2022; Malkiewicz et al., 2023).

Our findings and those of the previous studies (Risacher et al., 2016; Meng et al., 2022; Mur et al., 2023) highlight the importance of evaluating anticholinergic burden for cognitive function and brain function in older people. However, in our cohort, the effects were mostly not strong enough to be visible against the effects of AD-related neurodegeneration on subsequent cognitive decline and brain atrophy. It is worth noting that the level of anticholinergic burden was relatively low in our cohort, with most participants remaining at their initial burden level over time. This could reflect the highly selective nature of the memory clinic participants, who were mostly highly educated individuals with above-average health status. The effects may be more pronounced in less selective, population-based samples that represent a broader range of somatic diseases and polypharmacy (Kilimann et al., 2022). A previous study found higher anticholinergic burden associated with a higher risk of subjective cognitive decline in patients from a geriatric outpatient clinic, representing people with overall higher levels of somatic diseases and polypharmacy than the current cohort (Yavuz Veizi et al., 2025).

As a methodological note, using the Bayesian framework allowed for direct estimation of the strength of evidence for or against an effect. For the main effect of anticholinergic burden on CDR global score and basal forebrain volume the Bayes factors indicated evidence of no effect; for digit span, TMT-B and hippocampus volume it indicated inconclusive evidence with values around one. By design, based on the frequentist *p*-value one cannot quantify the evidence for absence of an effect as the *p*-value is calculated under the assumption that the null hypothesis is true. In frequentist inference, the null hypothesis is treated as a fixed assumption for the purpose of calculating the probability of the observed data—not as a probabilistic belief. Therefore, the frequentist *p*-value cannot provide a probability *for* or *against* the null. An inconclusive Bayes factor suggests that the data are uninformative for distinguishing between the alternative and null hypotheses due to a small sample size or trivial effect size. This is in contrast to frequentist *p*-values, where a non-significant p-value cannot distinguish between insufficient data and evidence supporting the null hypothesis.

As a shortcoming, the classical Bayes factor is sensitive to the prior distributions of the model parameters (O'Hagan, 1995), which can render the Bayes factor unreliable. Therefore, we based our analysis on a previously introduced cross-validated Bayes factor that relies on data splitting and is insensitive to choices of parameter priors (Hart and Malloure, 2019). When we checked the sensitivity of the cross-validated Bayes factor, we found it relatively stable between moderately and mildly informed priors, and consistent with the results of the parameter estimates for the effects of interest. This advantage came at the cost of a higher computational burden.

A strength of our study was the large sample of people with SCD, a group that has not often been studied in relation to anticholinergic burden and brain atrophy, and the longitudinal assessment of brain atrophy. One limitation was the highly selective cohort, which resulted in an overall low level of anticholinergic burden, and limits the generalization to the broader population of geriatric patients. Another limitation was the use of self-reports to assess medication use which could lead to exposure misclassification and be subject to recall bias. Additionally, we cannot be sure if patients were adherent to their medication. Another source of bias is protopathic bias, i.e., medication is initiated or discontinued in response to symptoms of a disease that is not yet diagnosed. We would not expect that anticholinergic treatment would be initiated in response to a symptom of a still undiagnosed cognitive disease, however, discontinuing anticholinergic treatment in response to a symptom of a still undiagnosed cognitive disease would be clinically plausible. This would then act against our primary hypothesis that anticholinergic burden increases risk of cognitive decline and brain atrophy. Finally, indication bias needs to be considered. Conditions associated with cognitive decline, such as behavioral impairment, may trigger the use of medication with anticholinergic burden. We cannot exclude this for our data, however, the majority of our participants were in very early stages of disease, like SCD, with little behavioral symptoms.

In summary, we found only limited evidence of an association between anticholinergic burden and cognitive performance in a cohort covering the entire AD spectrum, but evidence that there was no effect on basal forebrain and hippocampal volume. Based on previous findings, these volumes were expected to be sensitive to anticholinergic side effects. We suggest two factors might have contributed to the limited effects. First, we looked at a large number of cases, but they were from a highly selective cohort with relatively low anticholinergic burden. In addition, the presence of significant prodromal or clinical neurodegeneration may have masked the effects of anticholinergic burden, which may be more subtle than those of AD-related neurodegeneration. Nevertheless, it is noteworthy that some effects on cognitive measures were observed both at baseline and longitudinally. The lack of an effect on the basal forebrain and hippocampus may motivate data-driven studies of associations between anticholinergic burden and atrophy in other brain regions.

## Data Availability

The datasets presented in this article are not readily available because formal requests for data and biomaterials must be submitted to the DZNE Clinical Research Platform. Requests to access the datasets should be directed to klinische-studien@dzne.de.
