# Supplementary material for: Associations of anticholinergic burden of medication with cognitive decline and longitudinal brain atrophy in the Alzheimer’s disease spectrum
Source: Front Aging Neurosci. 2026 Mar 3;18:1751326. doi: 10.3389/fnagi.2026.1751326 (PMC12992049; doi:10.3389/fnagi.2026.1751326)
Supplement: Supplementary file 1 [file Data_Sheet_1.docx]

**Supplementary Table 1: Parameter estimates with mildly informed prior N(0, 1)**

| **Outcome** | **Parameter** | **Estimate [95% CI]** |
| --- | --- | --- |
| pacc5 | time | -0,617 [-0,7238 - -0,5124] |
| pacc5 | antichol. burden | -0,0727 [-0,1306 - -0,0147] |
| pacc5 | CN | 3,3182 [3,1137 - 3,5151] |
| pacc5 | MCI | 1,7738 [1,5656 - 1,9841] |
| pacc5 | Rel | 3,2537 [3,0113 - 3,4851] |
| pacc5 | SCD | 3,0836 [2,8915 - 3,2747] |
| pacc5 | sex | -0,4588 [-0,5544 - -0,3637] |
| pacc5 | education | 0,0746 [0,0584 - 0,0912] |
| pacc5 | time:antichol. burden | -0,0012 [-0,0188 - 0,0164] |
| pacc5 | time:CN | 0,665 [0,5588 - 0,7726] |
| pacc5 | time:MCI | 0,4204 [0,3085 - 0,5321] |
| pacc5 | time:Rel | 0,6276 [0,5125 - 0,7433] |
| pacc5 | time:SCD | 0,5928 [0,4874 - 0,6999] |
| CDR | time | 1,7675 [1,6302 - 1,9033] |
| CDR | antichol. burden | 0,0873 [0,0082 - 0,1652] |
| CDR | CN | -3,9277 [-4,1543 - -3,6937] |
| CDR | MCI | -2,5041 [-2,7478 - -2,2573] |
| CDR | Rel | -3,7133 [-3,9985 - -3,4212] |
| CDR | SCD | -3,6828 [-3,8977 - -3,463] |
| CDR | sex | -0,0201 [-0,1501 - 0,1115] |
| CDR | education | -0,0259 [-0,048 - -0,0037] |
| CDR | time:antichol. burden | -0,0106 [-0,0567 - 0,0354] |
| CDR | time:CN | -1,7475 [-1,8969 - -1,5953] |
| CDR | time:MCI | -1,3116 [-1,4746 - -1,149] |
| CDR | time:Rel | -1,7408 [-1,9245 - -1,5584] |
| CDR | time:SCD | -1,7201 [-1,8642 - -1,5745] |
| digit span total | time | -0,539 [-0,7632 - -0,316] |
| digit span total | antichol. burden | -0,0783 [-0,3072 - 0,1467] |
| digit span total | CN | 1,4887 [0,9773 - 1,9883] |
| digit span total | MCI | 0,6358 [0,1012 - 1,168] |
| digit span total | Rel | 1,277 [0,6446 - 1,8999] |
| digit span total | SCD | 1,481 [1,0118 - 1,9436] |
| digit span total | sex | -0,3604 [-0,7073 - -0,0132] |
| digit span total | education | 0,248 [0,1872 - 0,3091] |
| digit span total | time:antichol. burden | -0,0214 [-0,0937 - 0,0489] |
| digit span total | time:CN | 0,6565 [0,4192 - 0,8924] |
| digit span total | time:MCI | 0,3662 [0,1061 - 0,6251] |
| digit span total | time:Rel | 0,6409 [0,3495 - 0,9273] |
| digit span total | time:SCD | 0,5902 [0,3543 - 0,8241] |
| TMT-B | time | 1,5055 [0,7689 - 2,2454] |
| TMT-B | antichol. burden | 0,3928 [-0,5679 - 1,352] |
| TMT-B | CN | -0,4169 [-1,3842 - 0,5515] |
| TMT-B | MCI | 0,3138 [-0,6638 - 1,2824] |
| TMT-B | Rel | -0,1571 [-1,1379 - 0,8226] |
| TMT-B | SCD | -0,4067 [-1,3776 - 0,5609] |
| TMT-B | sex | 0,1287 [-0,8368 - 1,0871] |
| TMT-B | education | -1,9261 [-2,7131 - -1,1375] |
| TMT-B | time:antichol. burden | 0,8018 [0,0514 - 1,5491] |
| TMT-B | time:CN | -0,1904 [-1,0464 - 0,6692] |
| TMT-B | time:MCI | 0,9645 [0,0341 - 1,9177] |
| TMT-B | time:Rel | -0,1248 [-1,0658 - 0,8196] |
| TMT-B | time:SCD | 0,2014 [-0,6466 - 1,047] |
| basal forebrain | time | -0,0057 [-0,009 - -0,0024] |
| basal forebrain | antichol. burden | -0,0011 [-0,0043 - 0,0021] |
| basal forebrain | CN | 0,0722 [0,0623 - 0,0819] |
| basal forebrain | MCI | 0,0425 [0,0321 - 0,0529] |
| basal forebrain | Rel | 0,0748 [0,063 - 0,0866] |
| basal forebrain | SCD | 0,0635 [0,0543 - 0,0728] |
| basal forebrain | sex | -0,0098 [-0,015 - -0,0049] |
| basal forebrain | education | 0,0001 [-0,0007 - 0,001] |
| basal forebrain | time:antichol. burden | 0,0003 [-0,0007 - 0,0013] |
| basal forebrain | time:CN | 0,0054 [0,0019 - 0,0089] |
| basal forebrain | time:MCI | 0,0013 [-0,0025 - 0,0051] |
| basal forebrain | time:Rel | 0,0033 [-0,0007 - 0,0074] |
| basal forebrain | time:SCD | 0,0038 [0,0003 - 0,0072] |
| hippocampus | time | -0,1571 [-0,1751 - -0,1392] |
| hippocampus | antichol. burden | -0,02 [-0,0619 - 0,0219] |
| hippocampus | CN | 1,21 [1,0849 - 1,3357] |
| hippocampus | MCI | 0,6433 [0,5151 - 0,7731] |
| hippocampus | Rel | 1,2293 [1,0733 - 1,3863] |
| hippocampus | SCD | 1,0823 [0,9675 - 1,1964] |
| hippocampus | Sex | -0,2914 [-0,3601 - -0,224] |
| hippocampus | Education | 0,0066 [-0,0055 - 0,0185] |
| hippocampus | time:antichol. burden | 0,0027 [-0,003 - 0,0084] |
| hippocampus | time:CN | 0,1226 [0,1032 - 0,142] |
| hippocampus | time:MCI | 0,0478 [0,0268 - 0,069] |
| hippocampus | time:Rel | 0,1118 [0,0891 - 0,1345] |
| hippocampus | time:SCD | 0,1048 [0,0859 - 0,124] |

**Supplementary Figure 1: Anticholinergic burden trajectories by baseline octiles**

**
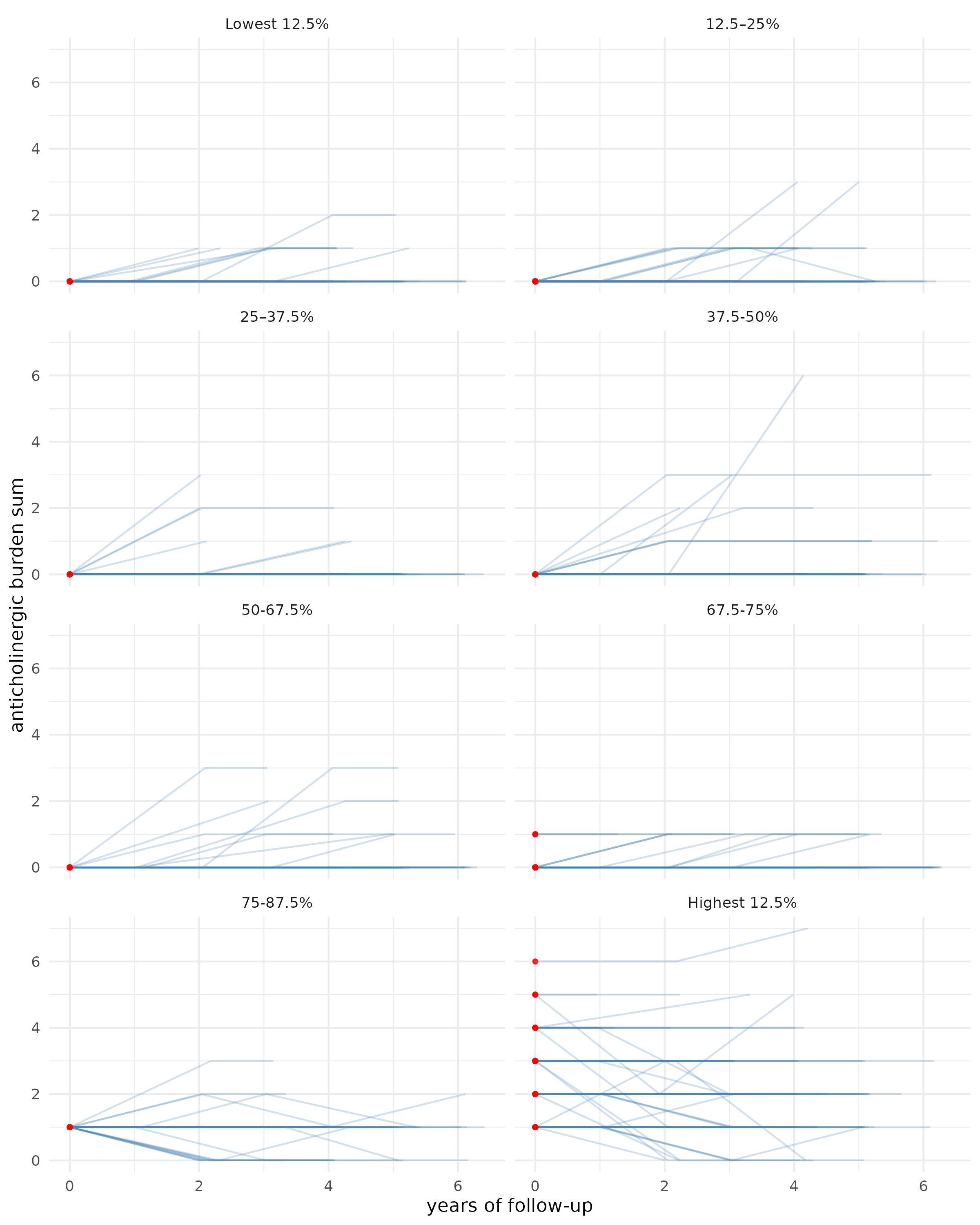
**

Individual trajectories of change in anticholinergic burden score over time of follow-up. For better visibility the sample has been split according to 12.5% quintiles of anticholinergic burden sum score at baseline (from lowest 12.5% upper left to highest 12.5% lower right).

**Supplement A: Annotated R-code for calculating cross-validated Bayes factors**

# Load required packages

library(brms) # Bayesian regression modeling using Stan

library(cmdstanr) # Backend for fitting Stan models via CmdStan

library(tidyverse) # Data manipulation and visualization

library(matrixStats) # Efficient matrix operations (e.g., logSumExp)

library(DescTools) # Additional statistical tools

# Define a function to compute cross-validated Bayes Factors (BF)

# number of iterations here is set to 50 (n_split),

# proportion of training vs. test sample is 0.85 (prop_train)

compute_cv_bf <- function(outcomes, data, n_splits = 50, prop_train = 0.85, n_boot = 2000, seed = 123) {

set.seed(seed) # Ensure reproducibility

results <- list() # To store results for each outcome

ids <- unique(data$ID) # Extract unique subject IDs (assumes 'ID' is the subject identifier)

n_ids <- length(ids) # Number of unique subjects

train_size <- round(prop_train * n_ids) # Number of subjects in training set per split

# Define prior (optional, if used in model);

# brms default flat priors are typically not recommended for assessing model fit

# here, we use a moderately informed prior

priors <- c(

prior(normal(0, 0.5), class = "b") # Normal prior for regression coefficients

)

# Loop over each outcome variable

for (outcome in outcomes) {

message("Running outcome: ", outcome) # Print status

log_score_m1 <- c() # Store log scores for Model 1 (with interaction)

log_score_m2 <- c() # Store log scores for Model 2 (without interaction)

# Perform repeated data splits for cross-validation

for (s in 1:n_splits) {

cat(" Split", s, "\n")

# Randomly sample training IDs; test set is the remaining

train_ids <- sample(ids, train_size)

test_ids <- setdiff(ids, train_ids)

# Create training and test data frames

df_train <- data %>% filter(ID %in% train_ids)

df_test <- data %>% filter(ID %in% test_ids)

# Define models: M1 includes time × antichol_burden interaction; M2 does not

formula1 <- as.formula(paste0(outcome, " ~ time*antichol_burden + time*diagnosis + sex + education + (1 + time | ID)"))

formula2 <- as.formula(paste0(outcome, " ~ antichol_burden + time*diagnosis + sex + education + (1 + time | ID)"))

# Fit both models using brms (Bayesian estimation)

fit1 <- brm(formula1, data = df_train, prior = priors,

warmup = 2000, iter = 8000, chains = 4, refresh = 0)

fit2 <- brm(formula2, data = df_train, prior = priors,

warmup = 2000, iter = 8000, chains = 4, refresh = 0)

# Compute log-likelihoods on test data

loglik1 <- log_lik(fit1, newdata = df_test, allow_new_levels = TRUE)

loglik2 <- log_lik(fit2, newdata = df_test, allow_new_levels = TRUE)

# Compute log predictive density (LPD) via log-sum-exp trick

lpd1 <- colLogSumExps(loglik1) - log(nrow(loglik1)) # For M1

lpd2 <- colLogSumExps(loglik2) - log(nrow(loglik2)) # For M2

# Append LPDs (pointwise log scores) to storage vectors

log_score_m1 <- c(log_score_m1, lpd1)

log_score_m2 <- c(log_score_m2, lpd2)

}

# Average log Bayes factor across splits

log_cv_bf <- (sum(log_score_m1, na.rm = TRUE) - sum(log_score_m2, na.rm = TRUE)) / n_splits

cv_bf <- exp(log_cv_bf) # Convert to Bayes factor scale

# Difference in log scores per split

log_diff <- log_score_m1 - log_score_m2

# Bootstrap 95% confidence interval for log BF

set.seed(123)

boot_sums <- replicate(n_boot, sum(sample(log_diff, replace = TRUE), na.rm = TRUE) / n_splits)

ci_log_cv_bf <- quantile(boot_sums, c(0.025, 0.975)) # CI on log scale

ci_cv_bf <- exp(ci_log_cv_bf) # CI on Bayes factor scale

# Store results for this outcome

results[[outcome]] <- list(

summary = tibble(

outcome = outcome,

cv_bf = cv_bf,

ci_cv_bf_lower = ci_cv_bf[1],

ci_cv_bf_upper = ci_cv_bf[2]

),

log_scores = list(

log_score_m1 = log_score_m1,

log_score_m2 = log_score_m2

)

)

}

# Combine all outcome summaries into a single table

result_table <- bind_rows(lapply(results, function(x) x$summary))

# Return summary table and raw log scores for downstream use

return(list(

summary = result_table,

log_scores = lapply(results, function(x) x$log_scores)

))

}

# Specify outcomes to test (replace with actual variable names)

outcomes <- c("outcome_1", "outcome_2", "outcome_n")

# Run cross-validated Bayes factor computation

cv_results <- compute_cv_bf(outcomes, data = data)

# View summary Bayes factor table

result_table <- cv_results$summary

print(result_table)

# Access raw log scores (e.g., for diagnostics or plotting)

log_scores_list <- cv_results$log_scores

str(log_scores_list) # Show structure of log scores list
